# CRISPR/Cas9 editing of *CBP80* enhances drought tolerance in potato (Solanum tuberosum)

**DOI:** 10.3389/fpls.2025.1598947

**Published:** 2025-07-10

**Authors:** C. A. Decima Oneto, G. A. Massa, L. Echarte, M. F. Rey Burusco, M. N. Gonzalez, C. S. Alfonso, M. P. Laserna, N. S. Norero, S. B. Divito, S. E. Feingold

**Affiliations:** ^1^ Laboratorio de Agrobiotecnología, Estación Experimental Agropecuaria (EEA) Balcarce-Instituto de Innovación para la Producción Agropecuaria y el Desarrollo Sostenible (IPADS) Unidad de Estudios Agropecuarios y Desarrollo de la Innovación Tecnológica Agropecuaria (UEDDINTA)–Consejo Nacional de Investigaciones Científicas y Técnicas (CONICET), Instituto Nacional de Tecnología Agropecuaria (INTA), Balcarce, Argentina; ^2^ Facultad de Ciencias Agrarias, Universidad Nacional de Mar del Plata, Balcarce, Argentina; ^3^ Comisión de Investigaciones Científicas de la Provincia de Buenos Aires (CIC), Tolosa, Argentina

**Keywords:** abiotic stress, abscisic acid, climate change, cap binding proteins, genome edited plants

## Abstract

Developing drought-tolerant potato varieties is increasingly important due to climate change and water scarcity, as potatoes are highly sensitive to water deficits that can significantly reduce yield and tuber quality. The cap-binding protein CBP80, involved in the abscisic acid (ABA) signalling pathway, has emerged as a promising target for improving drought tolerance in plants. In this study, we used CRISPR/Cas9 to edit the *StCBP80* gene in the tetraploid potato cultivar Spunta. Given the complexity of editing all four alleles in a tetraploid genome, eight independent partially edited lines (two or three alleles edited) were obtained. Two of these lines were selected for detailed molecular and phenotypic characterization. Under restricted water conditions, the selected lines exhibited reduced transpiration rates and improved leaf area index compared to non-edited controls. Gene expression analysis by quantitative real-time PCR showed differential expression of drought-responsive genes (P5CS, PDH, and *MYB33*), supporting a role for *StCBP80* in stress response modulation. Moreover, the edited lines showed lower yield penalties, both in biomass and tuber production, under drought stress. This work represents one of the first applications of genome editing to enhance drought tolerance in a commercial potato cultivar, and highlights CBP80 as a promising target for crop improvement. These findings provide valuable insights for the development of stress-resilient potato varieties using genome editing approaches.

## 1 Introduction

As extreme weather events become more frequent, improving crop resilience is essential to ensure stable production and food security. Enhancing drought tolerance through conventional breeding, molecular tools, and genome editing offers a sustainable solution to maintain productivity while optimizing water use efficiency. This is especially relevant in regions where irrigation resources are limited or becoming increasingly costly.

Understanding the molecular mechanisms underlying drought adaptation is essential for advancing plant breeding. The cap-binding protein 80 (CBP80), also known as Abscisic Acid Hypersensitive 1 (*ABH1*) in *Arabidopsis thaliana*, is a key regulator of the abscisic acid (ABA) signaling pathway and plays a crucial role in drought tolerance. CBP80, together with CBP20, forms the cap-binding complex (CBC), which binds to RNA polymerase II transcripts in the nucleus, ensuring mRNA stability, splicing efficiency, and miRNA biogenesis (Kierzkowski et al., 2009; Bartel et al., 2004; Reyes and Chua, 2007). In *Arabidopsis*, *AtCBP80* inactivation leads to ABA-hypersensitive stomatal closure, reducing wilting and improving drought resistance (Hugouvieux et al., 2001, 2002; Kmieciak et al., 2002). This response helps maintain turgor and enhances recovery after rewatering (Sinclair, 2017).

CBP80 has also been linked to the regulation of ABA-responsive genes, including *P5CS*, *PDH*, and the transcription factors *MYB33*, *MYB65*, and *MYB101*, which collectively contribute to drought adaptation by modulating stomatal responses and enhancing ABA sensitivity. Since ABA modulates *P5CS* and *PDH*, key genes in proline metabolism, CBP80 may influence proline accumulation and stress tolerance (Papp et al., 2004). Proline acts as an osmoprotectant, maintaining osmotic balance and preventing oxidative damage under water deficit. *P5CS* catalyzes proline biosynthesis, while *PDH* facilitates its degradation; under drought, increased *P5CS* expression and reduced *PDH* activity promote proline accumulation, enhancing stress resilience (Yang et al., 2021; Liu et al., 2019).

Additionally, *MYB33* is a positive regulator of ABA responses, particularly in stomatal function and seed germination. In *Arabidopsis*, its downregulation reduces stomatal sensitivity to ABA, weakening drought tolerance, whereas its overexpression enhances ABA sensitivity, improving stress adaptation (Wyrzykowska et al., 2022). *MYB33* is also post-transcriptionally regulated by microRNA159 (miR159), which fine-tunes its expression under drought conditions (Pieczynski et al., 2013; Alves et al., 2021).

The importance of the CBC in drought tolerance has also been demonstrated in crop species. For instance, in barley (*Hordeum vulgare*), mutation of the *HvCBP20* gene led to enhanced drought tolerance at both phenotypic and transcriptomic levels (Daszkowska-Golec et al., 2017). The *HvCBP20* mutant exhibited increased water retention, reduced stomatal conductance, and faster activation of stress-preventive mechanisms, highlighting the broader significance of CBC-mediated regulation in plant drought responses. Notably, in a highly water-sensitive crop as potato (Solanum tuberosum L.), silencing the *StCBP80* gene has shown to enhance stomata closure at comparable ABA concentration and delay turgor loss (Pieczynski et al., 2013).

Genome editing using the CRISPR/Cas9 system is a transformative tool for crop improvement, enabling the addition or modification of traits in many economically significant plant species (Arora and Narula, 2017; Baltes et al., 2017; Scheben et al., 2017; Gao, 2018). In its simplest application, the CRISPR/Cas9 system is used for targeted mutagenesis, where the Cas9 nuclease is guided to a specific genomic site by a single guide RNA (sgRNA), to introduce double-stranded breaks (DSBs) (Jinek et al., 2012). Mutations are induced in the target site in the form of small insertions or deletions (indels) upon repair by the error-prone non-homologous end joining (NHEJ) mechanism. When occurring within exons, such mutations can disrupt gene function (Puchta, 2005). This technology has been successfully applied in various crops such as maize, bread wheat, and potato, showcasing its versatility and efficiency (Svitashev et al., 2016; Liang et al., 2018; Andersson et al., 2018; González et al., 2020, Massa et al., 2025).

Moreover, CRISPR/Cas9 offers a significant advantage because the editing machinery does not require stable integration of foreign DNA into the plant genome (Woo et al., 2015; Gonzalez et al., 2021). This method aligns well with regulatory criteria in countries like Argentina and USA, among others (Fernandes et al., 2024), where genome-edited crops that do not contain foreign DNA are considered under similar regulation as conventionally bred varieties (Whelan and Lema, 2015; Lema, 2019). Traditional plant breeding methods are often labor-intensive and time-consuming, involving multiple cycles of crossing and selection. Non integrating gene editing offers an effective tool for incremental improvement in clonal crops (such as potatoes, bananas, cassava, sugarcane, and grapes) allowing for specific variability introduction in already established and adapted genotypes (Tiwari et al., 2020; Nahirñak et al., 2022).

CRISPR/Cas9 technology has previously been applied in potato to enhance various traits, including disease resistance (Norouzi et al., 2024; Poudel, 2024), improved nutritional quality (Das Dangol et al., 2024), and alterations in tuber color (Wulff-Vester et al., 2024). Zahid et al. (2024) enhanced stress resilience in potato by deleting Parakletos (a thylakoid protein) through CRISPR. Furthermore, one study demonstrated the potential of CRISPR in mitigating abiotic stress by modifying ABA metabolism in rice (Ye et al., 2023), yet no similar efforts have been reported in potato to date. This advancement is particularly significant given potato’s high sensitivity to water deficits and its crucial role as a global food crop (Nasir et al., 2022).

*StCBP80* has been previously silenced in potato using RNA interference, which resulted in enhanced drought tolerance through increased ABA sensitivity and stomatal regulation (Pieczynski et al., 2013). However, to date, no studies have employed CRISPR/Cas9 genome editing to achieve a knockout of the *StCBP80* gene. This represents a promising strategy, as CRISPR/Cas9 allows for precise and heritable modifications, enabling a deeper exploration of CBP80’s functional role in drought tolerance and its potential in crop improvement. In the present study, eight independent partially edited lines were obtained using CRISPR/Cas9, harboring mutations in two or three alleles but no complete (tetra-allelic) knockouts. We focus on the molecular and phenotypic characterization of two of these lines, selected based on detailed mutational analysis.

## 2 Materials and methods

### 2.1 SgRNA design on CBP80 gene of S. tuberosum cv. Spunta and vector assembly

**Figure 1:**
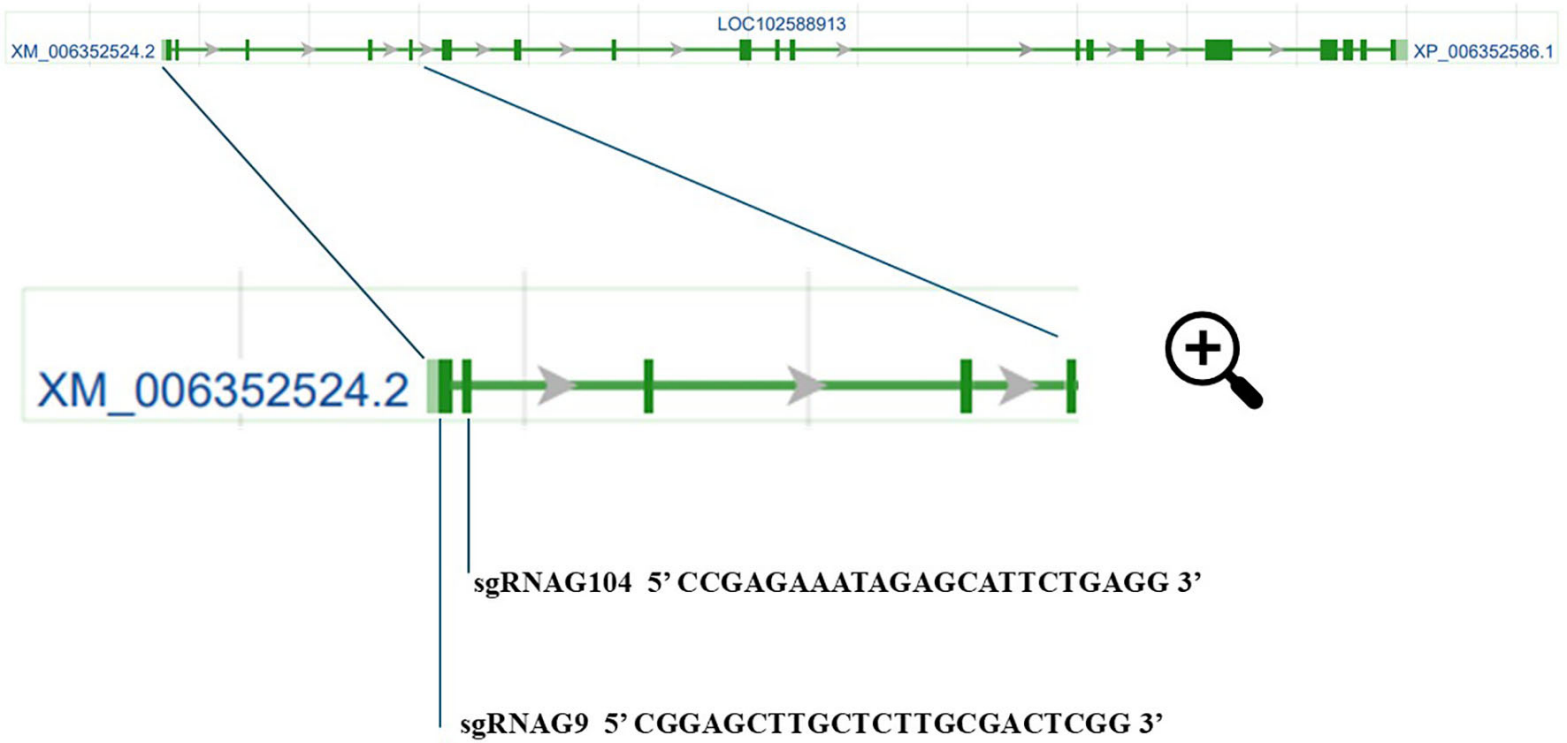
Schematic representation of the *StCBP80* gene of Solanum tuberosum (potato), which is composed of 19 exons interspersed with intronic regions. In this simplified representation, each “Exon” is depicted as a box, and each “Intron” is shown as a line separating the exons. The lengths of the boxes and lines are not proportional to the actual sequences but are designed to illustrate the gene’s structural organization. A zoomed-in view of the first four exons is shown, highlighting the design site of two sgRNAs: sgRNAG9 (exon 1), and sgRNAG104 (exon 2). These sgRNAs were strategically positioned for CRISPR/Cas9-mediated editing to disrupt the *StCBP80* gene function. The information on the gene structure and sgRNA locations was obtained from the NCBI database (https://www.ncbi.nlm.nih.gov/gene/102588913).

The available sequence of *Solanum tuberosum CBP80* gene (LOC102588913, gene description: nuclear cap-binding protein subunit 1, Andrade Díaz, 2024) was used for designing primers to amplify the *StCBP80* gene in the cultivar Spunta. Primers F1_StCBP80 (5’AATGAGTAGTTGGCGGAGCTT3’) and R10_StCBP80 (5’TACAGAGGTATCTTGTGAGGCA3’) amplified an expected 1,500 bp fragment from the 5′ region of the target gene, using 10 ng of genomic DNA as a template in a reaction with Phusion High-Fidelity DNA Polymerase (Thermo Fisher Scientific). Reaction conditions were 95°C for 1 min, 30 cycles of 95°C for 1 min, 55°C for 1 min, 72°C for 1 min and a final extension of 72°C for 10 min. PCR products were cloned into the pGEM^®^-T Easy Vector Systems (Promega) and transformed into One Shot TOP10 Chemically Competent E. coli (Thermo Fisher Scientific), according to the manufacturer´s instructions. Twelve randomly picked colonies were selected for plasmid purification and Sanger sequencing using the T7 and SP6 primers. The resulting sequences were aligned to avoid allelic variation during sgRNA design and for further High-Resolution Fragment Analysis (HRFA) primer design. The web-based tool a Cas-Designer Tool (http://www.rgenome.net/cas-designer/) was used for sgRNA design, using one of the sequences obtained for *StCBP80* as a query and S. tuberosum (PGSC v4.03) as a target genome. sgRNAG9 and sgRNAG104 (**Figure 1**) were selected according to the Out of Frame Score (Park et al., 2015) and the strict absence of allelic variation along the target sequence. The gRNAs were cloned into the pTRANS_100 vector under the Arabidopsis thaliana U6 promoter, following the Golden Gate system protocol developed by the Voytas group (Nadakuduti et al., 2019). This vector includes the coding sequence of the Cas9 nuclease protein under the control of the constitutive 35S promoter. This process yielded the final vector containing both sgRNAs: pTRANS_100G9-G104.

### 2.2 Protoplasts transfection and plant regeneration

The procedures for protoplast isolation and transfection were conducted according to previously described protocols (Nicolia et al., 2015, 2020; González et al., 2020). For protoplast isolation, 1 g of leaves from 5-week-old *in vitro* cv. Spunta platelets were used. The leaves were first incubated in Conditioning Medium and cell wall digestion was performed using an Enzymatic Solution composed of Cellulase Onozuka R-10 (Yakult Pharmaceutical) and Macerozyme R-10 (Yakult Pharmaceutical) in a medium osmotically balanced for the cells. The digestion involved incubating the plant tissue with the enzymatic solution for 14 hours in darkness at 25°C. After digestion, the protoplast suspension was filtered through a sterile nylon mesh with a pore size of 70 µm and purified using a 0.43 M Sucrose Solution. For transfections, 100,000 protoplasts were incubated with pTRANS_100G9G104 Vector and 40% Polyethylenglycol (PEG) 4000 (Duchefa Biochemie, Haarlem, The Netherlands) for 30 min. A regeneration control was included, which consisted of the same number of protoplasts incubated with 40% PEG without vector, for 30 min. After transfections, all protoplasts were embedded in sodium alginate and cultured for calli regeneration, according to Nicolia et al. (2015). Once protoplast calli were green and visible to the naked eye (0.5-1.0 mm diam.; 32-64 cell size) were released from alginate blobs (approximately after 33 days of culture) and subcultured for shoot growth induction. To ensure the analysis of independent lines, one shoot was picked per callus and transferred for root development.

### 2.3 Identification and genotypic analysis of CBP80-edited potato lines

Genomic DNA of regenerated plants was extracted from leaves according to Haymes, 1996. The detection of putative mutations in the target gene was performed by High Resolution Fragment Analysis (HRFA), according to Andersson et al. (2018). The primer CBPHRFA-Rev, labelled with Fluorescein Amidite (FAM), was designed and used in combination with the primer F1_*StCBP80* to amplify a 200 bp fragment of the CBP gene, encompassing both sgRNA target sites within the *StCBP80* gene. PCR was carried out using Q5^®^ High-Fidelity DNA Polymerase (New England Biolabs) under the following reaction conditions (98°C for 5 min, 34 cycles of 98°C 1 min s, 55°C 1 min, 72°C 30 s, and a final extension of 72°C for 10 min). PCR product length was determined by GeneMarker Software v3.0.1 (SoftGenetics, State College, PA, USA) and insertions or deletions were identified comparing peak presence/absence or displacement comparing line electropherograms versus the non-edited control. Target regions of *StCBP80* gene were sequenced by Next Generation Sequencing service (NGS; Celemics, Republic of Korea) in selected edited lines to confirm HRFA results. Sequencing results were analyzed with Geneious Software v2023.1.2 (https://www.geneious.com/) and insertions or deletions were identified comparing each line versus the non-edited control.

### 2.4 Experiments for assessing water deficit responses

Two types of water deficit experiments were conducted. The first one aimed to evaluate transpiration response and water use efficiency under controlled soil water deficits (water deficit experiment). The second one was focused on assessing differences in soil water thresholds during progressive soil drying (drying soil experiment).

#### 2.4.1 Water deficit experiment

Selected *in vitro*-regenerated plants were transferred to a substrate on 10 L pots in a greenhouse at temperatures ranging between 20°C and 27°C with a photoperiod of 16 hours light (120 µmol m−2 s−1) and 8 hours dark. Surface of the pots was sealed with plastic bags to avoid evaporation. Ten biological replicates were grown for each edited line and the non-edited control line Spunta under a normal irrigation regime. After four weeks, irrigation was discontinued for half of the pots of all genotypes. Soil water content in the pots was maintained at 30% of field capacity for 37 days. Pot water content was measured with a moisture meter previously calibrated (Bluelab Pulse Meter). After water deficit period, irrigation was restored to 100% field capacity. The remaining biological replicates were kept at 100% field capacity, throughout the entire experiment.

#### 2.4.2 Relative expression of genes

Relative gene expression quantification of *StCBP80*, Pyrroline-5-Carboxylate Synthetase (P5CS), and Proline Dehydrogenase (PDH) was performed using real-time PCR. *P5CS* and *PDH* genes were selected due to their well-documented roles in molecular stress responses through proline biosynthesis and degradation. *StCBP80* expression was analyzed in both edited and control lines. Elongation Factor 1-Alpha (*EF1α2*) was used as the reference gene for normalization in all qPCR assays. Total RNA was extracted using TRIzol reagent (Invitrogen) from flash-frozen leaves collected on day 37 after drought onset in both treatments and stored at -80°C. cDNA synthesis was performed using the PrimeScript RT reagent kit (Takara). Specific primers for qPCR for each target gene were designed (**Table 1**). Primer efficiency was calculated to ensure accurate quantification. Real-time PCR was conducted using a 7500 Applied Biosystems real-time cycler with SYBR Green detection. Relative expression levels of the target genes were quantified using the ΔΔCt method (Livak and Schmittgen, 2001) For each sample, the Ct values of the target genes were normalized to the elongation factor reference gene to obtain the ΔCt. The ΔΔCt was then calculated by comparing the ΔCt of the treated samples to that of the control samples. The relative expression levels were expressed as 2^(-ΔΔCt). Statistical analysis of the ΔΔCt values was performed using ANOVA to determine the significance of gene expression differences between edited and control lines under both irrigation regimes. For this analysis, five plants were used for the CBP80-39 and the non-edited while four plants were used for the CBP80-32 genotype. In all cases, three technical replicates were performed for each sample.

**Table 1:** Primer sequences used for quantitative PCR (qPCR) analysis of gene expression.

Gene name	Forward primer	Reverse primer	Annealing temperature (°C)	References
*EF1α 2*	CTTGACGCTCTTGACCAGATT	GAAGACGGAGGGGTTTGTCT	60	Campbell et al., 2023
*P5CS*	CTCCAAGCGATCCACAATCA	GTCATACCACCTCTTCCAACTC	59	Designed in this study
*PDH*	TGGTATGGCAGATGGTCTTTC	CAGCACGCCTCATAAGGTAAT	60	Designed in this study
*CBP80*	TTCTTCAACCCTCGTCCTTTAC	GTGATTGACAGTTATACGGGAGAT	60	Designed in this study
*MYB33*	ATGAGCATCACAAGTGAAACCG	CTACACGGCTGACATGGCATCCCA	60	Wyrzykowska et al., 2022

The table lists the forward and reverse primer sequences, their corresponding annealing temperatures (°C), and their sources.

#### 2.4.3 Daily transpiration rate and cumulative transpiration

The daily transpiration rate (ml d-1) was determined weekly from the onset of the drought period as the difference in pot weight between consecutive measurements divided by the time between measurements (usually 24 hours). Weekly transpiration rates were aggregated, and the cumulative transpiration was calculated by summing weekly values from the start of the water deficit period until harvest.

#### 2.4.4 Yield and Water use efficiency

After the completion of the water stress period and at the time of harvest, all tubers from each plant were carefully collected and separated. The fresh weight of the tubers was immediately recorded using a precision balance. Additionally, the total number of tubers per plant was counted. Water use efficiency was estimated as the quotient between yield and cumulative transpiration.

#### 2.4.5 Biomass

Biomass was determined after harvesting the tubers by first weighing the fresh above-ground parts of the potato plants. These samples were then dried at 70°C for 15 days to reach a constant weight. The dry weight of each sample was subsequently recorded. Above-ground biomass calculations were based on the dry weight of the vegetative plant material.

#### 2.4.6 Canopy cover

Canopy cover, defined as the percentage of ground area covered by plant leaves, was assessed using the CANOPEO application (Patrignani and Ochsner, 2015). This mobile tool estimates green canopy cover by analyzing the proportion of green pixels in digital images. Images were taken from directly above each individual plant at a fixed height of 50 cm using a smartphone camera with standard settings and under consistent ambient light conditions to minimize variability. To avoid edge effects, plants located at the borders of the experimental layout were excluded from canopy cover measurements. Only plants positioned in the inner rows were considered. The canopy cover percentage was calculated automatically by the CANOPEO app, which identifies green vegetation based on pixel color thresholds. Measurements were conducted at multiple time points: before stress onset, during the drought period, and after rewatering, under both well-watered and water-deficit conditions, for both edited and non-edited plants. The same protocol was consistently applied throughout the experiment to ensure comparability across treatments and time points.

#### 2.4.7 Stomatal density

Stomatal density was measured on fully expanded leaves of the second node. Five biological samples of 30-day-old potato plants were sampled for the analysis. The abaxial leaf surface peels were obtained using transparent adhesive tape and mounted on glass slides. Images were observed under a light microscope at 10x magnification and captured using a Leica camera attached. Stomatal density was calculated as the number of stomata per unit area (stomata/mm²). For each leaf, five random fields of view were analyzed, and the average stomatal density was recorded by counting the number of stomata per image with an area of 1.288 mm^2^.

#### 2.4.8 Statistical analysis

Statistical analyses were performed using InfoStat (Di Rienzo et al., 2020), a software that integrates R as its computational engine, specifically utilizing R version 3.6.2 for statistical procedures and data analysis. Initially, a normality test was conducted for all data sets corresponding to each evaluated condition. If the data exhibited a normal distribution, an ANOVA test was performed, followed by a Tukey’s *post-hoc* test (α=0.05). For data sets that did not meet the normality assumption, a non-parametric Kruskal-Wallis test was applied (α=0.05).

### 2.5 Drying soil experiment

Selected *in vitro*-regenerated plants were transferred to 1 L pots with substrate and placed in a growth chamber at a constant temperature of 24°C with a photoperiod of 16 hours light (270 µmol m−2 s−1) and 8 hours dark. Ten biological replicates were grown for each edited line and the non-edited control line Spunta under a normal irrigation regime. After 3 weeks, irrigation was stopped in five replicates of each genotype. On the afternoon before the start of the experiment, the pots were overwatered and allowed to fully drain. To prevent evaporation directly from the substrate, the pots were sealed with plastic bags. The initial weight of each pot was recorded at the beginning of the photoperiod the following morning. Pots were weighed twice daily, approximately at the start and the midpoint of the daylight period. Transpiration rate during the daylight hours was calculated as the difference in pot weight divided by the elapsed time between measurements. The transpiration rate for each pot was normalized (NTR) by dividing the individual transpiration measurement by the average transpiration rate of three well-irrigated replicates for each genotype. On each day of the dry-down experiment, the water content of the root media was calculated as the fraction of transpirable soil water (FTSW). The total transpirable soil water in each pot was calculated as the difference between the initial weight of the pot and the pot weight when NTR ≤ 0.10.

#### 2.5.1 Statistical analysis

The NTR values were analyzed as a function of FTSW using a two-segment linear regression model (GraphPad Prism 7). The regression output included the FTSW value for the intersection between the two linear segments (i.e., FTSW threshold) and the 95% confidence interval for the intersection.

## 3 Results

### 3.1 Generation and characterization of CBP80-edited potato lines

To investigate the role of CBP80 in drought tolerance, we employed CRISPR/Cas9 to introduce targeted mutations in the *StCBP80* gene of the tetraploid potato cultivar Spunta. Our aim was to generate stable knockouts that could help elucidate CBP80’s function in stress responses and assess whether its disruption could enhance drought tolerance. By targeting conserved coding regions with two specific guide RNAs (sgRNA-G9 and sgRNA-G104), we sought to induce frameshift mutations leading to loss of function, ultimately evaluating the physiological and agronomic impact of *StCBP80* inactivation in potato.

The CRISPR/Cas9 system was successfully employed to edit the *StCBP80* gene of the tetraploid potato cultivar Spunta. HRFA and NGS confirmed successful editing in alleles of several regenerated lines. Two edited lines, CBP80-32 and CBP80-39, were selected for further analysis based on the type of mutations they carried, and the number of edited alleles detected by amplicon-based NGS. In CBP80-32, two distinct edited alleles were detected: one containing a single nucleotide deletion with a frequency of 21.6%, and another with a single nucleotide insertion at 26.6%, out of a total of 1,680 reads. In CBP80-39, three different edited alleles were identified: two independent single nucleotide deletions with frequencies of 20.7% and 23.5%, respectively, and one single nucleotide insertion at 19.3%, from a total of 1,940 reads.

**Figure 2:**
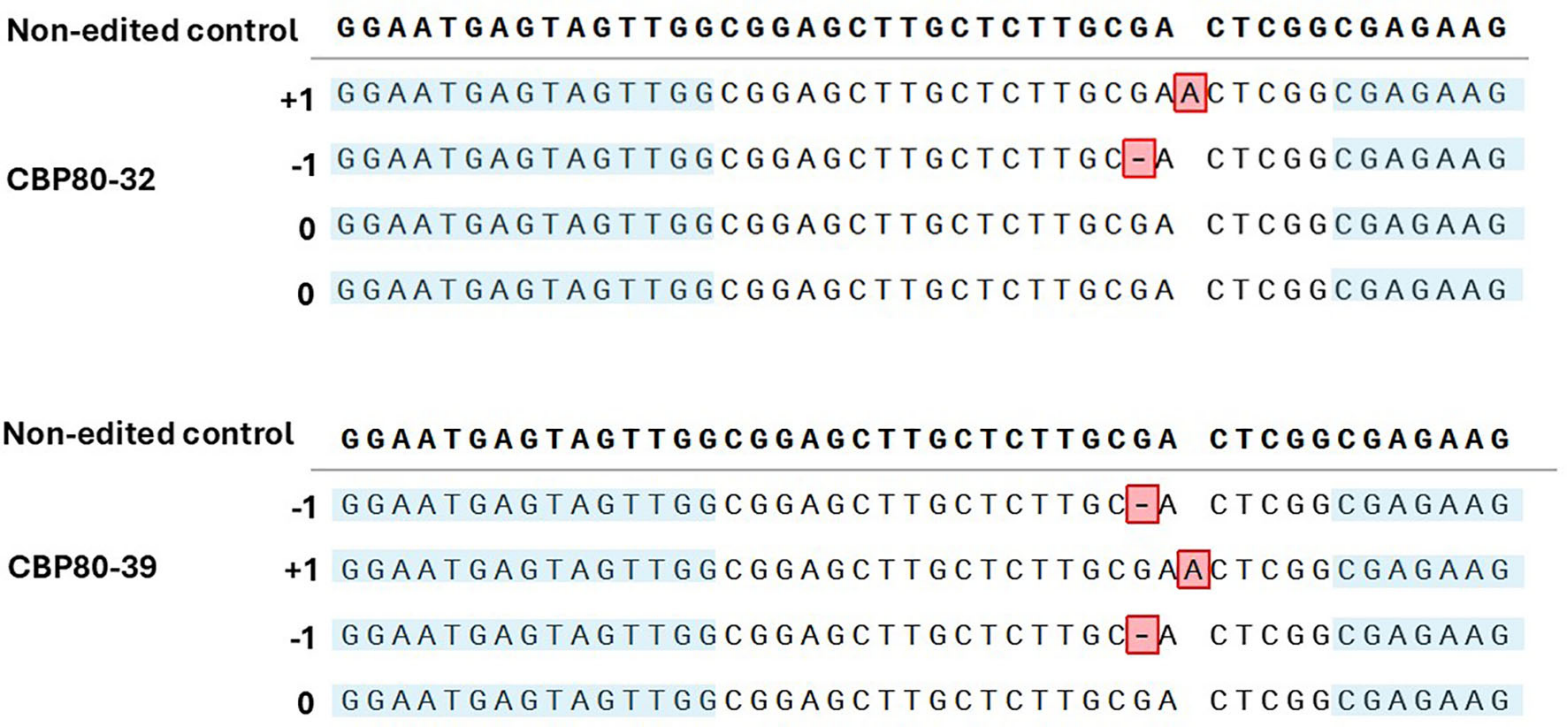
Sequences highlighted in blue indicate the nucleotide sequences of the non-edited control *StCBP80* gene and the edited lines CBP80-39 and CBP80-32 at the sgRNA-G9 target site. Mutations detected in the edited lines include a single-base insertion (A) in one allele and two independent single-base deletions (G) in the CBP80-39 line. In the CBP80-32 line, a single-base deletion (G) and a single-base insertion (A) were detected in two distinct alleles.

Based on these early evaluations, CBP80-32 and CBP80-39 were chosen as the most promising candidates for subsequent physiological and molecular characterization. Mutations in most lines consisted predominantly of small insertions or deletions (indels) leading to frameshift mutations. In both selected edited lines, mutations were exclusively detected at the sgRNAG9 target site. In the CBP80-39 edited line, two types of mutations were identified across three alleles: a single-base insertion (A) in one allele and a single-base deletion (G) in two alleles (**Figure 2**).

### 3.2 Relative expression of *StCBP80* and drought-related genes

Quantitative real-time PCR (qPCR) was performed to measure the expression levels of the *StCBP80*, *PDH*, *P5CS*, and *MYB33* genes. Statistical analyses were conducted using unpaired, two-tailed Student’s t-tests with Welch’s correction for unequal variances, applying a significance threshold (alpha) of 0.05.

The expression of *StCBP80* was assessed in two edited lines (CBP80-39 and CBP80-32) and the non-edited control under both well-watered (WW) and water-deficit (WD) conditions.

**Figure 3:**
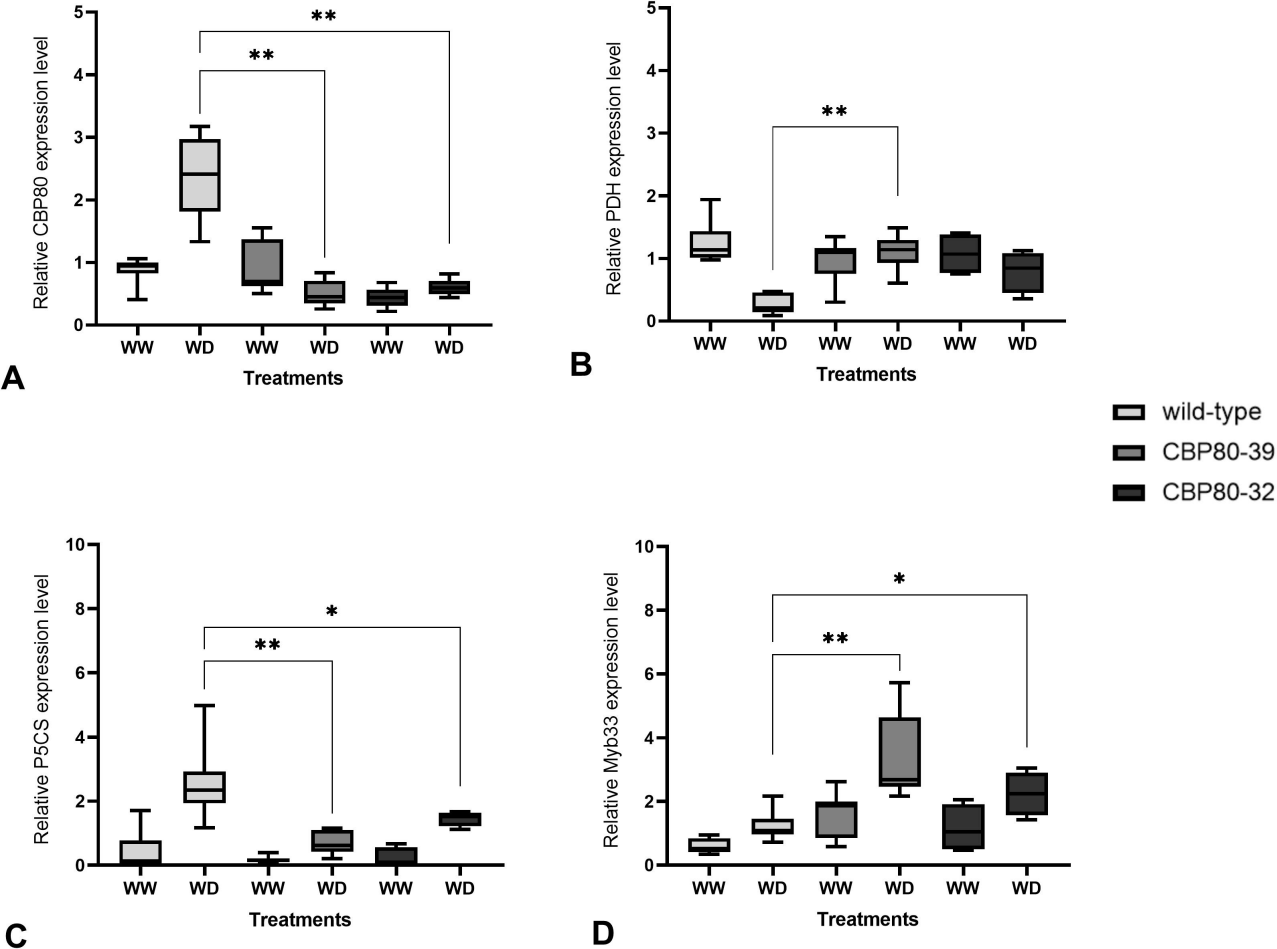
Relative expression levels of *StCBP80* **(A)**, PDH **(B)**, P5CS **(C)**, and *MYB33* **(D)** in the non-edited control and the edited lines (CBP80-32 and CBP80-39) under well-watered (WW) and water-deficit (WD) conditions. Statistical analyses were conducted using unpaired, two-tailed Student’s t-tests with Welch’s correction for unequal variances, applying a significance threshold (alpha) of 0.05. The comparisons presented are: non-edited control WD versus CBP80-39 WD, and non-edited control WD versus CBP80-32 WD. A single asterisk (*) denotes statistically significant differences (p < 0.05), while a double asterisk (**) indicates highly statistically significant differences (p < 0.01). Five biological samples were used for each genotype and condition, with three technical replicates per sample.

In the non-edited control, *StCBP80* expression was significantly higher under WD compared to WW, with an approximate twofold increase (p < 0.001; **Figure 3A**).

In contrast, expression levels in CBP80-39 and CBP80-32 remained relatively stable across conditions.

Under WD, both CBP80-39 (p = 0.0001) and CBP80-32 (p = 0.0002) exhibited significantly lower *StCBP80* expression compared to the non-edited control.

*PDH* expression was significantly reduced in the non-edited control under WD compared to WW (p < 0.001; **Figure 3B**). Higher *PDH* expression was detected in CBP80-39 under WD compared to the non-edited control (p < 0.001). A borderline significant difference was observed between CBP80-32 and the non-edited control under WD (p = 0.0502), suggesting a trend towards increased *PDH* expression in CBP80-32.

*P5CS* expression was significantly upregulated in the non-edited control under WD relative to WW (p = 0.0002; **Figure 3C**). In CBP80-39 and CBP80-32, *P5CS* expression remained relatively unchanged between treatments. Compared to the non-edited control under WD, both CBP80-39 (p = 0.00067) and CBP80-32 (p = 0.0166) exhibited significantly reduced *P5CS* expression.

Under WD conditions, *MYB33* expression showed an increase.

In CBP80-39, *MYB33* expression was approximately fourfold higher than in the non-edited control (p = 0.0014; **Figure 3D**). A statistically significant increase was also detected in CBP80-32 under WD compared to the non-edited control (p = 0.042), although this result should be interpreted with caution due to its proximity to the significance threshold. The smaller increase in *MYB33* expression in CBP80-32 relative to CBP80-39 may be related to differences in the allelic dosage of the non-edited *StCBP80*. We focused our analysis on *MYB33*, which, despite its high homology with *MYB65* and *MYB101* (Millar and Gubler, 2005), has been identified as the most responsive to miR159-mediated post-transcriptional regulation, and as a key regulator in drought stress responses (Allen et al., 2007; Reyes and Chua, 2007). Moreover, *MYB33* expression has consistently been reported as dependent on *CBP80/ABH1* in *Arabidopsis thaliana* and *Solanum tuberosum* (Pieczynski et al., 2013), further supporting its selection for this study.

### 3.3 Phenotypic evaluation under drought stress

#### 3.3.1 Canopy cover

To assess the impact of *StCBP80* editing on drought tolerance, canopy cover was evaluated as an indicator of plant growth and water stress response. Canopy cover, defined as the percentage of ground area covered by plant leaves, was measured using the CANOPEO application (Patrignani and Ochsner, 2015), a non-destructive tool that estimates green canopy cover based on digital image analysis. Measurements were taken before, during, and after the drying period under two irrigation regimes: 100% (well-watered) and 30% (water deficit) field capacity.

**Figure 4:**
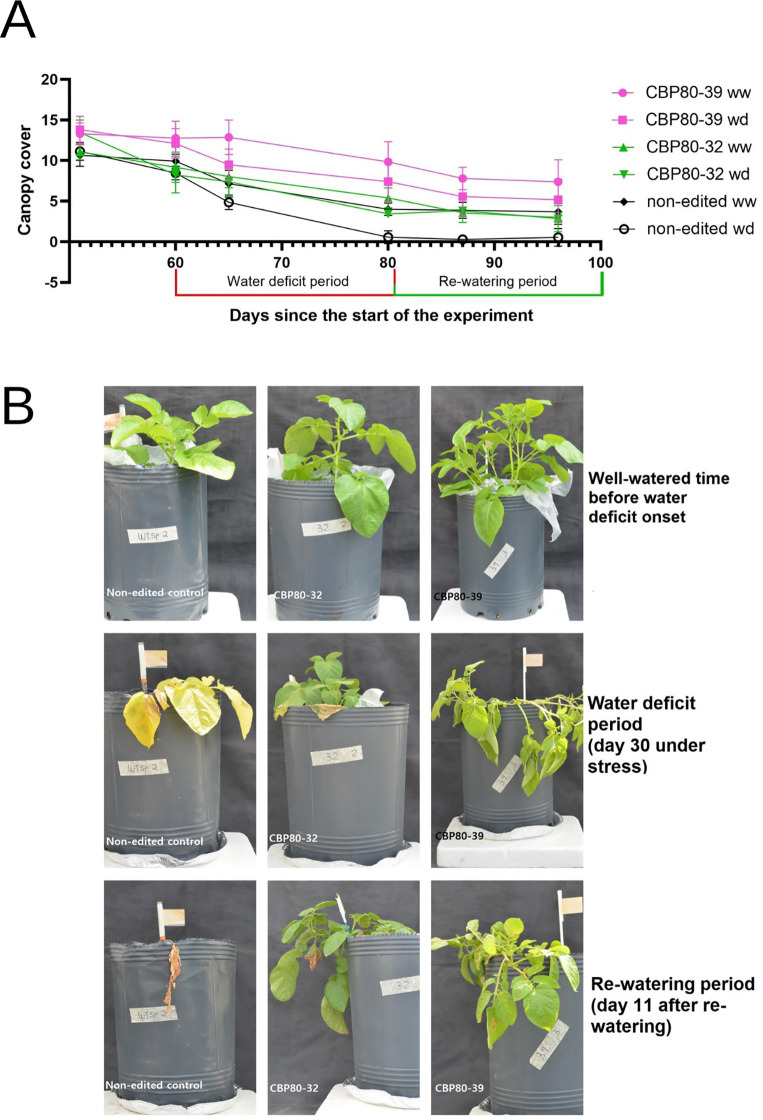
**(A)** Temporal variation of canopy cover for edited lines (CBP80-32 and CBP80-39) and the non-edited control under two water regimes: well-watered (WW) and water-deficit (WD). The water-deficit phase occurred between days 50 and 80, followed by rewatering from day 81 onwards. Data represent mean ± standard error for each treatment (n= 5 plants per genotype and water condition). **(B)** Temporal variation of canopy cover for edited lines (CBP80-32 and CBP80-39) and the non-edited control during the assay.

The canopy cover measurements revealed significant differences between the edited lines and the non-edited control among irrigation regimes over time (**Figure 4A**). Under WD, the edited lines CBP80-39 and CBP80-32 consistently maintained higher green canopy coverage compared to the non-edited control. For instance, on day 96 since the beginning of the assay, CBP80-39 under WD retained an average canopy of 6.07, while CBP80-32 maintained 3.56, both significantly higher than the wild-type, which fell to 0.75. Comparing the canopy cover progression within genotypes, edited line CBP80-39 showed a decline from 12.18 (WW, day 60) to 6.07 (WD, day 96), representing a 50% reduction, whereas edited line CBP80-32 exhibited a sharper decline from 9.36 (WW) to 3.56 (WD) in the same period, representing a 38% reduction. In contrast, the non-edited control demonstrated a nearly complete loss (>90%) of canopy cover under WD conditions, dropping from 3.21 (day 80) to 0.75 (day 96), indicating severe drought susceptibility (**Figure 4B**).

Overall, the edited lines CBP80-39 and CBP80-32 displayed greater resilience under WD compared to the non-edited control, suggesting that *StCBP80* editing enhances the ability to maintain canopy integrity during water-limited conditions.

#### 3.3.2 Transpiration rate and cumulative transpiration

**Figure 5:**
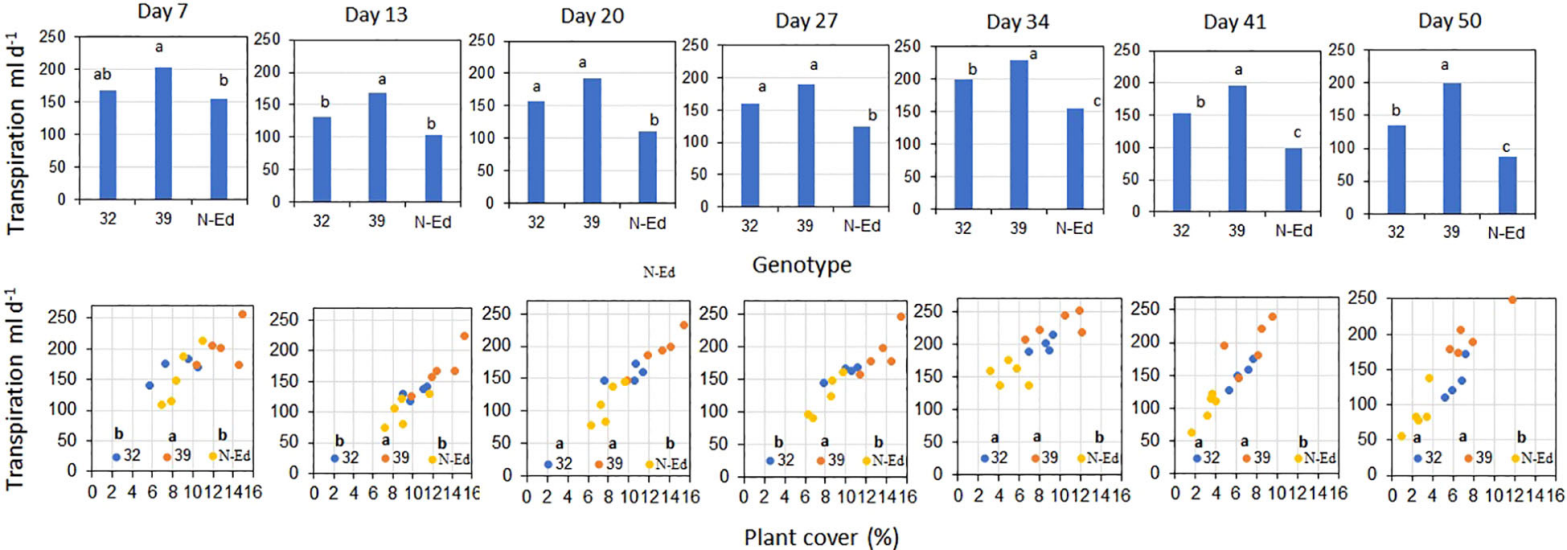
Transpiration rate as a function of (i) genotype (edited lines and non-edited control, upper panel) and of (ii) plant cover (%, lower panel) for well irrigated plants measured over the experimental period. 32: CBP80-32, 39: CBP80-39 and N-Ed: non-edited control. Different letters indicate significant differences statistically significant differences (p < 0.05). (n= 5 plants per genotype and water condition).

The transpiration rate was consistently higher in CBP80-39 compared to CBP80-32 and the non-edited control under well-watered (WW) conditions throughout the experimental period (p < 0.05; **Figure 5**). Higher transpiration rates were associated with greater plant canopy cover (**Figure 5**).

**Figure 6:**
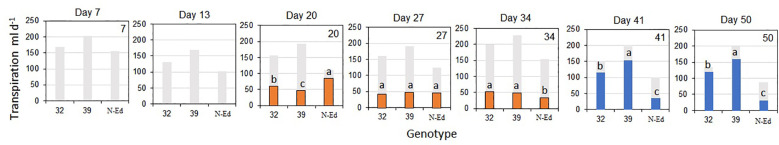
Transpiration rate as a function of genotype for water-limited plants during the drying period (orange bars) and after rewatering (blue bars). Different letters indicate significant differences among genotypes (p<0.05) in each day of measurement. Well-irrigated plants throughout the experiment are included as a reference (grey bars). 32: CBP80-32, 39: CBP80-39 and N-Ed: non-edited control. (n= 5 plants per genotype and water condition). No error bars are shown at days 7 and 13 for water-limited plants because transpiration measurements began only after the soil water content dropped to 30% of field capacity.

The transpiration rate was significantly lower under WD conditions compared to WW conditions within each genotype (p < 0.05). During the drying period, the mean reductions in transpiration rate were greater in the edited lines (69.3% for CBP80-32 and 76.2% for CBP80-32) compared to the non-edited control (57.2%; **Figure 6**).

After rewatering, the transpiration rate of edited lines increased to levels comparable to WW treatment (**Figure 6**). Cumulative transpiration after rewatering was closer to that of WW plants in CBP80-32 (17% lower than WW) and CBP80-39 (20% lower than WW) whereas it remained significantly lower in the non-edited control (63% lower than WW). Consequently, cumulative transpiration, including both the drying and post-rewatering periods, was significantly higher (p<0.05) in the edited lines (3.0 and 3.6 liters, for CBP80-32 and CBP80-39 respectively) than in the non-edited control (1.7 liters).

#### 3.3.3 Stomatal density

**Figure 7:**
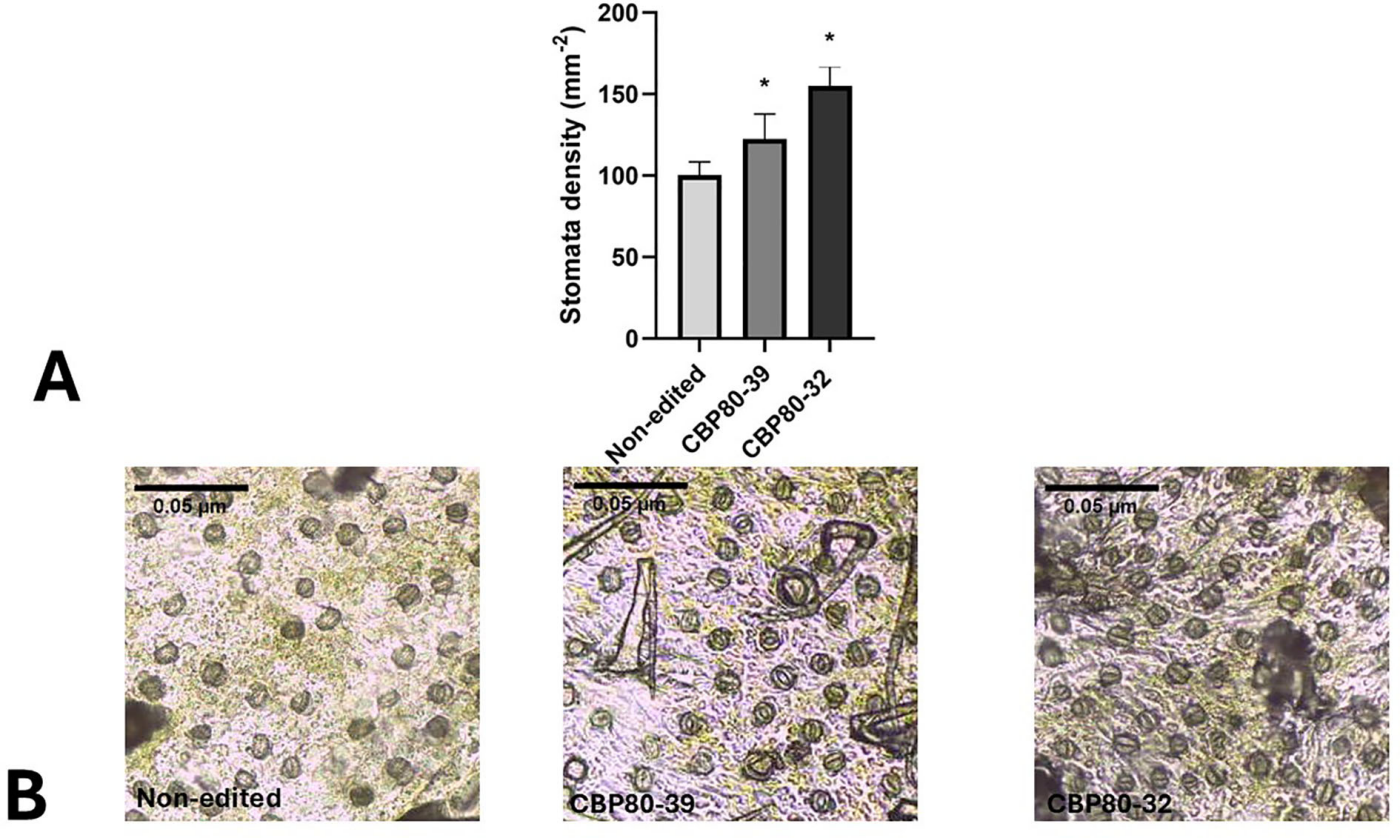
**(A)** Stomatal density in *StCBP80*-edited lines and non-edited control. The graph represents stomatal density (stomata mm^-2^) on the abaxial leaf surface of the *StCBP80*-edited lines (CBP80-32 and CBP80-39) and the non-edited control. Error bars indicate standard deviation. *Above the bars indicate statistically significant differences (p < 0.05). 32: CBP80-32, 39: CBP80-39 and N-Ed: non-edited control. **(B)** Representative microscopic images of stomata impressions.

Stomatal density analysis revealed significant differences among the edited lines and the non-edited control. Both edited lines, CBP80-32 and CBP80-39, exhibited a markedly higher number of stomata per unit area on the abaxial leaf surface compared to the non-edited control (**Figure 7A**). Specifically, CBP80-32 displayed the highest stomatal density, reaching an average of 150 stomata mm^-2^, followed by CBP80-39 with approximately 120 stomata mm^-2^. In contrast, the non-edited control maintained a significantly lower stomatal density, averaging 90 stomata mm^-2^ (**Figure 7B**).

#### 3.3.4 Normalized transpiration rate as a function of fraction of transpirable soil water

**Figure 8:**
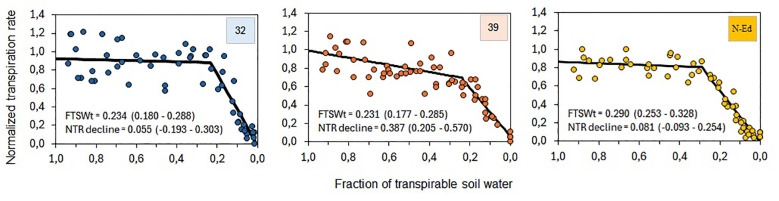
Normalized transpiration rate (NTR) as a function of the fraction of transpirable soil water (FTSW) for lines 32 and 39, and the non-edited control. The threshold of FTSW for the onset of NTR sharp decline (FTSWt) and the slope of NTR decline above FTSWt are indicated in each panel, with confidence intervals shown in brackets. 32: CBP80-32, 39: CBP80-32 and SP: non-edited control.

The normalized transpiration rate (NTR) as a function of the fraction of transpirable soil water (FTSW) reveals distinct responses among the *StCBP80*-edited lines (CBP80-32 and CBP80-39) and the non-edited control. The threshold FTSW (FTSWt), representing the point at which NTR sharply declines, was lower in CBP80-32 (0.234) and CBP80-39 (0.231) compared to the non-edited control (0.290). This indicates that the edited lines maintained higher transpiration rates under greater soil water depletion before reaching the threshold at which transpiration significantly dropped. Moreover, the slope of NTR decline above FTSWt was steeper in CBP80-39 (-0.387) compared to CBP80-32 (-0.055) and the non-edited control (-0.081), suggesting a more abrupt reduction in transpiration in this line once the threshold was reached (**Figure 8**).

#### 3.3.5 Yield, biomass and water use efficiency

**Figure 9:**
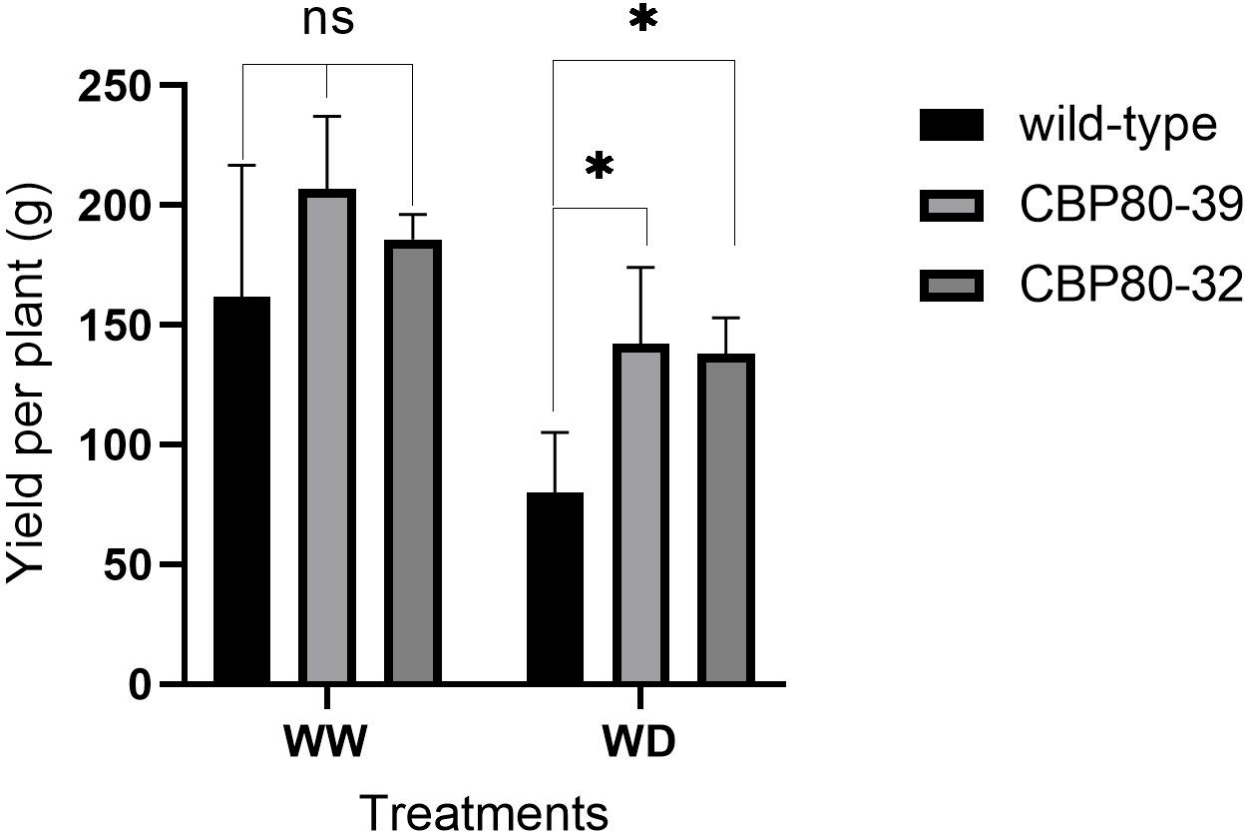
Tuber yield per plant (g) in the edited lines (CBP80-32 and CBP80-39) and the non-edited control under both well-watered (WW) and water-deficit (WD) conditions. * Above the bars indicate statistically significant differences (p < 0.05) and ns indicate non statistically significant differences (p ≥ 0.05), (n= 5 plants per genotype and water condition).

At harvest, the edited lines showed a significantly smaller decrease in both tuber yield and biomass under water deficit (WD) conditions compared to the non-edited plants (**Figure 9**). While yield decreased in all lines under WD relative to well-watered (WW) conditions, the reduction was less substantial in the edited lines than in the wild-type. Under WW conditions, tuber yield did not differ significantly between the edited lines (CBP80-32 and CBP80-39) and the wild-type. CBP80-32 yielded an average of 185.64 g per plant, while CBP80-39 produced 206.76 g per plant, with no statistical differences between them. However, the non-edited control showed a lower average yield of 139.31 g per plant, although this difference was not statistically significant (**Figure 9**). Under water-deficit (WD) conditions, all genotypes experienced a reduction in yield; however, the edited lines demonstrated better yield retention compared to the non-edited control. The non-edited control showed the greatest sensitivity to water deficit, with a 41.78% yield reduction from its well-watered (WW) yield. In contrast, the edited lines exhibited improved drought resilience, with CBP80-32 showing the smallest decline (27.75% from its WW yield) and CBP80-39 experiencing a 31.23% reduction from its WW yield (**Figure 9**).

#### 3.3.6 Yield and water use efficiency

**Figure 10:**
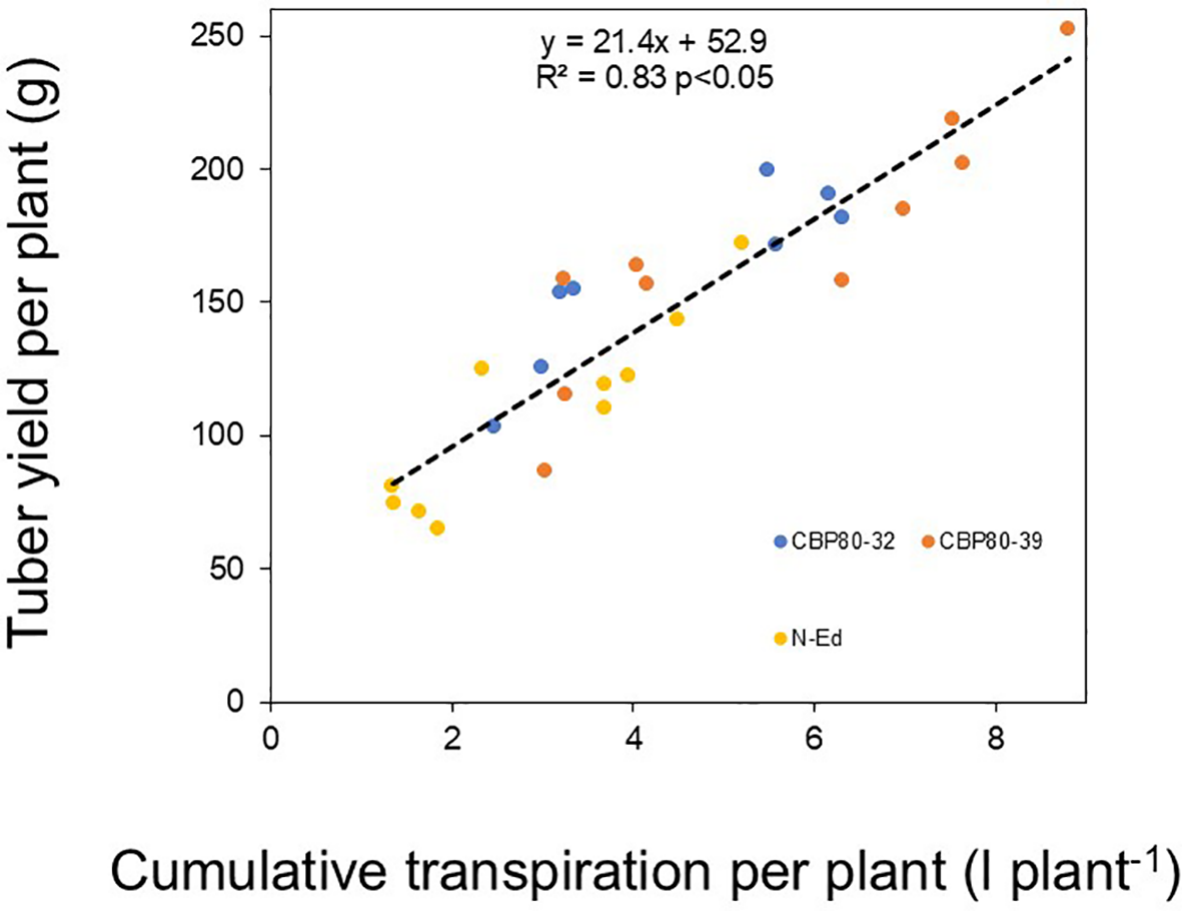
Yield per plant (g plant^-1^) as a function of cumulative transpiration per plant (L plant^-1^) in the non-edited control (yellow symbols) and the edited lines CBP80-32 (blue symbols) and CBP80-39 (orange symbols) across different water regimes.

Water use efficiency (calculated as the ratio of tuber yield to cumulative transpiration) under WW conditions was higher in CBP80-32 and non-edited control (32 g L^-1^) compared to CBP80-39 (27 g L^-1^; p < 0.05). Under WD conditions, no significant differences were observed among the edited lines and the non-edited control, with water use efficiency values of 44, 38, and 49 g L^-1^ for CBP80-32, CBP80-39, and the wild-type, respectively. Therefore, tuber yield differences across genotypes were closely related to cumulative transpiration differences (R2 = 0.83; p <0.05; **Figure 10**).

## 4 Discussion

This study provides further evidence of the role of *StCBP80* in drought tolerance in *Solanum tuberosum* cv. Spunta. The edited lines exhibited a multifaceted drought-resilience phenotype, including improved physiological and molecular traits, which collectively underscore the pivotal role of CBP80 in the plant’s drought response mechanisms.

The CRISPR/Cas9-mediated editing of the *StCBP80* gene in potato successfully introduced mutations in the form of single-base insertions or deletions (indels), resulting in expression alterations of this gene and other related ones. Specifically, the edited line CBP80-32 exhibited mutations in two alleles, while CBP80-39 displayed three mutated alleles. Notably, no large fragment deletions or full knockouts (mutations in all four alleles) were observed. The absence of full knockouts could be attributed to either limited efficiency of the selected sgRNAs, or to a critical role of CBP80 in plant development and survival, leading to lethality in lines with complete loss of function (Hugouvieux et al., 2001; Pieczynski et al., 2013). Similar findings have been reported in *Arabidopsis*, where *AtCBP80* has been shown to be essential for ABA signaling and stress responses (Hugouvieux et al., 2001). The methodological framework employed in this study is supported by recent advances in CRISPR/Cas9 applications in potato. González et al. (2023) developed robust protocols for both transgenic and transgene-free genome editing, optimising sgRNA design, vector assembly, RNP delivery, and regeneration of edited lines. These tools have greatly facilitated functional characterisation of target genes and accelerated precision breeding in complex tetraploid cultivars such as Solanum tuberosum. Apparently, the conservation of at least one wt allele was enough to assure viability in edited lines. Although the edited lines showed enhanced drought tolerance, it is important to acknowledge that partial editing of the *StCBP80* gene may result in residual CBP80 function. In both CBP80-32 and CBP80-39 lines, not all alleles were fully mutated. Furthermore, we recognize the potential influence of somaclonal variation associated with protoplast-derived regeneration. Although the use of non-edited control plants regenerated under identical *in vitro* and greenhouse conditions reduces the likelihood that the observed phenotypic differences are solely due to somaclonal effects, we cannot fully exclude this possibility.

Our approach contrasts with previous approaches that silenced *StCBP80* using RNA interference (RNAi). For example, Pieczynski et al. (2013) demonstrated enhanced drought tolerance in *StCBP80*-silenced potato lines; however, these lines remained transgenic and relied on artificial microRNAs. In contrast, CRISPR/Cas9 editing introduces stable, heritable modifications without foreign DNA integration (Woo et al., 2015). Additionally, silenced lines are prone to gene expression reversion, whereas the mutations introduced by CRISPR/Cas9 are permanent, ensuring stable phenotypic outcomes across generations. These findings underscore the advantages of genome editing over silencing techniques, particularly in its ability to achieve stable and precise genetic modifications, which can be considered regulatory equivalent to varieties derived from conventional breeding (Lema, 2019).

*P5CS*, a key enzyme in proline biosynthesis, has been extensively studied for its role in accumulating proline under water-deficit conditions, acting as an osmoprotectant in various plant species including potato and maize (Daszkowska-Golec et al., 2017; Teper-Bamnolker et al., 2023). Similarly, *PDH*, which is involved in the degradation of proline, is differentially regulated during stress to maintain proline as a critical osmoprotectant (Daszkowska-Golec et al., 2017). *MYB33* was selected based on its reported involvement in the molecular regulation of CBP80, particularly its role in miRNA biogenesis and stress response pathways in Arabidopsis (Hugouvieux et al., 2001; Reyes and Chua, 2007).

The differential expression of *StCBP80*, *P5CS*, and *PDH* in the edited lines suggests distinct mechanisms underpinning drought tolerance. *StCBP80* expression in the non-edited control (cv. Spunta) was significantly upregulated under water-deficit conditions, suggesting its involvement in the regulation of stress-responsive pathways. This aligns with findings in *Arabidopsis thaliana*, where *AtCBP80* knockouts displayed enhanced ABA sensitivity, leading to more efficient stomatal closure and reduced water loss (Hugouvieux et al., 2001). In contrast, the edited lines showed no induction of *StCBP80* expression under drought, suggesting functional disruption of the gene. The upregulation of *P5CS* shows a potential enhancement of proline biosynthesis in the drought response of all lines. Proline acts as an osmoprotectant, stabilizing cellular structures and scavenging reactive oxygen species (ROS) under stress (Szabados and Savouré, 2010; Kaur and Asthir, 2015). While the observed increase in *P5CS* expression is consistent with findings in other crops, such as maize and wheat, where enhanced proline accumulation correlates with improved drought tolerance (Zhang et al., 2022; Liang et al., 2018), proline concentration measurements are needed to determine if this response directly contributes to the increased drought resilience observed in *StCBP80*-edited lines. Conversely, *PDH* expression, involved in proline catabolism, was downregulated in both the non-edited and edited lines under drought conditions. This reduction likely reflects a conserved strategy to conserve proline during water stress, as seen in previous research on proline metabolism in plants (Verslues and Sharma, 2010). However, relative *PDH* expression levels in edited lines under WW conditions is significantly lower than in the non-edited line. This feature could be related to a higher basal proline concentration that may influence drought tolerance.

The most notable expression pattern change caused by the editing of *StCBP80* was observed in *MYB33*. This transcription factor showed higher expression levels under well-watered (WW) conditions in both edited lines, along with a strong induction under water-deficit (WD) conditions, which was not detected in the non-edited line. These expression trends support the hypothesis that *MYB33* may play a crucial role in the drought resilience of the edited potato lines, potentially through its interaction with ABA-mediated signaling pathways, as observed in *Arabidopsis*. These results are consistent with findings by Wyrzykowska et al. (2022), who demonstrated that overexpression of *MYB* transcription factors enhances ABA sensitivity and improves drought stress tolerance. Additionally, Pieczynski et al. (2013) reported that silencing of the CBP80 gene led to increased *MYB33* expression, further supporting its role in stress adaptation mechanisms.

Crop yield can be understood as the result of cumulative transpiration and water use efficiency for yield production (Echarte et al., 2023). In this context, our results demonstrate that the two edited lines outperformed the non-edited control in terms of (i) cumulative transpiration, in both the dry and post-rewatering periods, and (ii) tuber yield under both WW and WD conditions.

The relationship between normalized transpiration rate (NTR) and the fraction of transpirable soil water (FTSW) was effectively described by a two-segment linear model in both edited lines and the non-edited control, which is consistent with findings reported for other crops (Sadras and Milroy, 1996; Sinclair, 2017; Echarte et al., 2023). The FTSW threshold (FTSWt) for the sharp decline in NTR as FTSW decreased was within the range of values reported in these previous studies. However, a distinctive outcome of the present study was the lower FTSWt observed in the edited lines compared to the non-edited control. A lower threshold likely facilitates the maintenance of higher relative transpiration under low soil water availability, contributing to increasing cumulative transpiration in environments with transient water limitation. The higher fraction of transpirable soil water threshold (FTSWt) observed in the edited lines indicates that they can sustain transpiration for a longer period before experiencing a sharp decline, potentially allowing for more efficient water use under drought conditions. These findings suggest that *StCBP80*-edited lines exhibit a more conservative water-use strategy by sustaining transpiration under moderate soil water depletion while rapidly reducing water loss once a critical threshold is crossed. This response could enhance drought resilience by delaying the onset of severe water stress while optimizing water-use efficiency.

The more pronounced decrease in normalized transpiration rate (NTR) observed in CBP80-39 suggests that this line may have a stronger ability to regulate transpiration in response to declining soil moisture, potentially contributing to improved drought tolerance. Overall, these findings indicate that *StCBP80* editing influences plant water management, which may be a key factor in the observed drought resilience of the edited lines. These results suggest that *StCBP80*-edited lines exhibit an altered water-use strategy compared to the non-edited control.

The lower FTSW threshold observed in the edited lines indicates a more gradual decline in transpiration compared to the non-edited control, enhancing their ability to sustain transpiration under water-limited conditions. This finding aligns with the results of Pieczynski et al. (2013), who showed that in *StCBP80*-silenced lines, stomatal aperture was higher in the absence of ABA, lower at moderate ABA concentrations, and similar to the wild type at high ABA concentrations. In the current study, the gradual reduction in transpiration (i.e. in CBP80-39) and the lower FTSW threshold (i.e. in CBP80-32 and CBP80-39) observed in the edited lines may reflect a modulated stomatal response to ABA. Under water-limited conditions, where ABA levels are likely moderate, the edited lines may exhibit a more controlled stomatal closure. This fine-tuned stomatal behavior may enhance water management during stress periods, contributing to the higher cumulative transpiration observed in the edited lines under water-deficient conditions. In crops under irrigation, this feature can contribute to smarter water management, reducing irrigation frequency without compromising growth and yield.

The edited lines maintained significantly higher tuber yields and biomass under WD conditions compared to the non-edited control, reducing yield penalties. Pieczynski et al. (2013) demonstrated that silencing *StCBP80* expression in potato using artificial microRNAs resulted in significant physiological and morphological changes associated with drought tolerance. Transgenic plants exhibited ABA-hypersensitive stomatal closure, leading to a reduction in transpiration rates and improved water retention during drought conditions. Additionally, these plants showed increased stomatal density on the abaxial leaf surface, potentially enhancing their capacity for gas exchange under optimal conditions while maintaining a more controlled water loss during stress. These modifications collectively resulted in enhanced drought resilience, as evidenced by higher relative water content (RWC) and improved recovery upon rehydration compared to non-edited plants (Pieczynski et al., 2013). Interestingly, even under well-watered conditions, the edited lines outperformed the non-edited control in terms of yield, suggesting pleiotropic effects of *StCBP80* disruption on growth and productivity or a potential outcome related to experimental conditions.

CBP80, a key component of the cap-binding complex, plays a critical role in various cellular processes, including mRNA processing, translation, and stress responses. In plants, CBP80 has been implicated in regulating ABA signaling and drought tolerance. The downregulation of *StCBP80* in the edited potato lines led to enhanced drought tolerance, suggesting that *StCBP80* may act as a negative regulator of drought stress responses. This marked downregulation indicates a possible involvement of CBP80 in the plant’s adaptive response to water stress, potentially by enhancing the transcription of stress-responsive genes.

The observed increase in stomatal density in *StCBP80*-edited lines provides compelling evidence of a compensatory mechanism triggered by *StCBP80* downregulation, consistent with previous reports in *Arabidopsis* and potato (Pieczynski et al., 2013). While a higher stomatal density is traditionally associated with increased transpiration rates, it is crucial to consider the ABA hypersensitivity previously described in *AtCBP80*-mutant plants (Hugouvieux et al., 2001). This trait likely enables the edited lines to balance water loss through a more responsive stomatal closure mechanism, preventing excessive transpiration under drought conditions. Such a dynamic regulatory mechanism suggests that stomatal patterning in *StCBP80*-edited plants is not merely a structural adaptation but a functional one, enhancing their ability to optimize water loss and carbon assimilation depending on environmental conditions.

The relationship between stomatal density and water-use efficiency (WUE) is complex and highly dependent on environmental constraints. Increased stomatal density, coupled with ABA hypersensitivity, may enhance the capacity for rapid stomatal adjustments in response to fluctuating soil moisture, potentially improving WUE over time (Daszkowska-Golec et al., 2017). However, no changes in WUE were observed between the edited lines and the non-edited control. This regulation is particularly relevant when considering the superior drought resilience observed in the edited lines, as evidenced by their ability to maintain higher canopy cover and biomass accumulation under water-deficit conditions. A denser canopy contributes to a more favorable microclimate, reducing soil evaporation and further modulating plant water status. Additionally, the enhanced canopy observed in *StCBP80*-edited lines suggests that their increased stomatal density does not impose a significant growth penalty due to higher water consumption. Instead, it may contribute to sustaining photosynthetic capacity during periods of stress.

These findings reinforce the idea that CBP80 plays a dual role in regulating both stomatal density and stomatal responsiveness, positioning it as a key genetic target for engineering drought-tolerant potato cultivars. The ability of *StCBP80*-edited plants to integrate physiological and morphological responses (ranging from stomatal function to whole-plant water balance) highlights its potential for improving crop resilience under increasingly unpredictable climatic conditions.

Moreover, another mechanism by which CBP80 may influence drought tolerance is through its role in regulating the expression of stress-responsive genes. CBP80 has been shown to interact with various transcription factors and RNA-binding proteins, which can modulate the expression of genes involved in stress response (Daszkowska-Golec et al., 2017; Kim et al., 2021). By disrupting the function of *StCBP80*, the edited lines may have altered the expression of these genes, leading to enhanced drought tolerance. The CBP80 protein (when forming the CBC heterodimer) interact directly with mRNAs, controlling their maturation and processing. Some of these mRNAs might belong to different transcription factors (such as *MYB33*).

A recent study by Zahid et al. (2024) provided compelling evidence that deletion of *Parakletos*, a gene encoding a thylakoid-associated protein, enhances stress resilience in potato by modulating chloroplast function and photosynthetic performance. Using CRISPR, the authors demonstrated improved drought and salt tolerance, underscoring the relevance of targeting non-traditional loci to strengthen abiotic stress responses in potato. Our work complements these findings by focusing on a distinct regulatory pathway: we employed CRISPR/Cas9-mediated editing of CBP80, a nuclear cap-binding protein involved in ABA signaling and mRNA processing. Together, these studies highlight how diverse molecular targets—*Parakletos* in the chloroplast and CBP80 in the nucleus—can be leveraged to enhance drought tolerance through complementary biotechnological strategies in potato.

Finally, CBP80 is involved in ABA signaling. ABA is a key hormone involved in plant responses to various stresses, including drought. CBP80 has been shown to interact with several components of the ABA signaling pathway, including ABI1 and ABI2, which are negative regulators of ABA signaling (Hugouvieux et al., 2001). By disrupting *StCBP80* function, the edited lines may have enhanced ABA sensitivity, leading to improved drought tolerance.

### 4.1 Broader implications and future directions

This work reinforces the conserved role of CBP80 in plant drought responses, expanding previous findings in Arabidopsis to a tetraploid crop such as potato. Our results demonstrate the potential of CRISPR/Cas9-mediated genome editing as a precise and efficient approach for improving stress tolerance in crops. In countries with supportive regulatory frameworks, such as Argentina, the application of genome editing technologies may accelerate the development and commercialization of climate-resilient cultivars (Whelan & Lema, 2015; Lema, 2019).

Future research could apply transcriptomic and metabolomic analyses to unravel the broader molecular pathways influenced by CBP80 and their specific contributions to drought resilience. Comparative studies involving additional stress-related genes may further clarify the capacity of genome editing to modulate complex agronomic traits. At present, the edited potato lines are undergoing field trials to evaluate whether the enhanced drought tolerance observed under greenhouse conditions is maintained in open-field environments. Importantly, a formal consultation with the National Commission on Agricultural Biotechnology (CONABIA) confirmed that both CBP80-edited lines (CBP80-32 and CBP80-39) are not classified as transgenic.

Future research should further explore the interactions between stomatal traits, proline accumulation, ABA signaling, and hydraulic conductivity to refine breeding strategies aimed at maximizing both yield and stress tolerance in potato. Measuring proline levels in the edited lines will provide additional insights into the role of CBP80 in osmotic adjustment under drought conditions. Additionally, transcriptomic and metabolomic approaches could offer a deeper understanding of the molecular networks regulated by CBP80 and their contribution to drought resilience.

## 5 Conclusions

This study provides strong evidence that CBP80 plays a critical role in drought tolerance in *Solanum tuberosum* cv. Spunta. *StCBP80*-edited potato lines generated through CRISPR/Cas9 technology exhibited enhanced drought tolerance, characterized by physiological improvements such as elevated proline accumulation, reduced water loss through transpiration, and improved canopy stability under water-limited conditions. These phenotypic traits correlated with altered expression of drought-responsive genes, including *P5CS*, *PDH*, and *MYB33*, underscoring a regulatory role for CBP80 in stress signaling pathways. Furthermore, edited plants maintained higher tuber yields and biomass under drought stress, reinforcing the utility of genome editing for improving crop performance in challenging environments.

The observed water-deficit-tolerance phenotype in *StCBP80*-edited lines aligns with previous findings in *Arabidopsis thaliana* and barley, where mutations in CBP components enhanced abiotic stress adaptation. Notably, the edited potato lines maintained higher transpiration rates under moderate soil water depletion while rapidly reducing water loss once a critical threshold was reached. This altered water-use strategy, together with improved stomatal control, supports the role of CBP80 in integrating molecular and physiological drought responses. Although full gene knockout was not achieved, the partial editing of *StCBP80* preserved essential gene functions while still enhancing stress resilience, demonstrating the feasibility of using precise genome modifications to improve agronomic traits in a tetraploid crop.

Beyond the specific impact on potato drought tolerance, these findings contribute to a broader understanding of how cap-binding proteins mediate abiotic stress adaptation in plants. The regulatory link between CBP80 and *MYB33* suggests that CBP80 may influence ABA-dependent stress responses beyond stomatal regulation, potentially affecting transcriptional networks involved in drought signaling. Furthermore, the differential expression of *P5CS* and *PDH* in the edited lines highlights the relevance of CBP80 in proline metabolism, a crucial osmoprotective mechanism in plants. Future research should investigate how CBP80 interacts with other transcriptional regulators and RNA-processing pathways to further refine its functional role in drought adaptation.

Future studies should also explore the interactions between stomatal traits, proline accumulation, ABA signaling, and hydraulic conductivity to refine breeding strategies aimed at maximizing both yield and drought resilience in potato. Additionally, measuring proline levels in the edited lines will provide a more comprehensive assessment of osmotic adjustment mechanisms. Future studies will also include measuring ABA levels in the edited plants to better understand hormonal regulation under stress conditions. Expanding this research to other potato cultivars and related species could reveal whether *StCBP80* editing has a consistent impact across different genetic backgrounds. Furthermore, integrating transcriptomic and metabolomic analyses will help identify downstream targets of CBP80, offering new insights into the molecular networks governing stress adaptation.

In conclusion, this study demonstrates that the precise genome editing of *StCBP80* represents a promising strategy for improving drought tolerance in potato, a crop highly sensitive to water deficits. By enhancing key physiological traits, modulating stress-responsive gene expression, and maintaining yield under water-limited conditions, this approach provides a valuable tool for climate-tolerant agriculture. As extreme weather events become more frequent, harnessing genome editing to enhance stress resilience in crops will be essential to ensure global food security and sustainable agricultural production.

## Data Availability

The datasets presented in this study can be found in online repositories. The names of the repository/repositories and accession number(s) can be found in the article/supplementary material.
