# Correction: CRISPR/Cas9 editing of *CBP80* enhances drought tolerance in potato (Solanum tuberosum)

**DOI:** 10.3389/fpls.2025.1674362

**Published:** 2025-08-22

**Authors:** C. A. Decima Oneto, G. A. Massa, L. Echarte, M. F. Rey Burusco, M. N. Gonzalez, C. S. Alfonso, M. P. Laserna, N. S. Norero, S. B. Divito, S. E. Feingold

**Affiliations:** ^1^ Laboratorio de Agrobiotecnología, Estación Experimental Agropecuaria (EEA) Balcarce-Instituto de Innovación para la Producción Agropecuaria y el Desarrollo Sostenible (IPADS) Unidad de Estudios Agropecuarios y Desarrollo de la Innovación Tecnológica Agropecuaria (UEDDINTA)–Consejo Nacional de Investigaciones Científicas y Técnicas (CONICET), Instituto Nacional de Tecnología Agropecuaria (INTA), Balcarce, Argentina; ^2^ Facultad de Ciencias Agrarias, Universidad Nacional de Mar del Plata, Balcarce, Argentina; ^3^ Comisión de Investigaciones Científicas de la Provincia de Buenos Aires (CIC), Tolosa, Argentina

**Keywords:** abiotic stress, abscisic acid, climate change, cap binding proteins, genome edited plants

An affiliation “Comisión de Investigaciones Científicas de la Provincia de Buenos Aires (CIC), Tolosa, Argentina” was omitted for author “M. F. Rey Burusco”. This affiliation has now been added for author “M. F. Rey Burusco”.

In the published article, there was an error in the **Conflict of interest** statement. This sentence previously stated:

“The reviewer EA declared a shared affiliation with the authors MG and PL to the handling editor at the time of review.”

The corrected sentence appears below:

“The reviewer EA declared a shared affiliation with the author MG to the handling editor at the time of review.”

The original version of this article has been updated.

